# Increased antibody productivity in Chinese Hamster Ovary cells through induction of chromosomal instability by cell fusion

**DOI:** 10.1186/1753-6561-9-S9-P11

**Published:** 2015-12-14

**Authors:** YuanShan Lai, Noriko Yamano, Masayoshi Onitsuka, Takeshi Omasa

**Affiliations:** 1 Graduate School of Advanced Technology and Science, Tokushima University, Tokushima, Japan; 2 Institute of Technology and Science, Tokushima University, Tokushima, Japan; 3 Graduate School of Engineering, Osaka University, Osaka, Japan

## Background

Chromosomal instability, which often occurs as a random event in an uncontrollable manner, is a key characteristic of Chinese Hamster Ovary (CHO) cells. This often complicates the process of establishing stable, high antibody-producing CHO cell lines. We previously found that the abnormal increase of chromosome number is linked to increased antibody production in adherent CHO DG44 cells. In this study, we analyzed the relationship between chromosomal variety and antibody production in CHO K1-derived IgG1-producing cells by inducing cell fusion to artificially generate chromosomal variation within CHO DG44 cells.

## Materials and methods

Cell fusion in CHO K1-derived IgG1-producing cells was induced by polyethylene glycol (PEG). Efficiency of fusion was examined using red (CellTrackerRed CMPTX, Molecular Probes, Eugene, OR, USA) and blue (CellTrackerBlue CMAC, Molecular Probes, Eugene, OR, USA) fluorescent dyes and confirmed by flow cytometry. Five cell lines were selected from untreated and PEG-treated cells by single cell cloning using the ClonePix2 system (Molecular Devices, Sunnyvale, CA, USA) represented as clones NON-HYB-1-NON-HYB-5 and clones #1-#5, respectively, in this report. Each clone was stained with propidium iodide (PI) and the DNA content was measured using flow cytometry to determine their ploidy in comparison to the control (untreated) cell pool. DNA content was defined by DNA index (DI), the ratio of fluorescence intensity of control versus sample cells in G0/G1 phase. To further analyze the chromosome number distribution, chromosome numbers were determined for all the cell lines by microscopic observation of DAPI-stained metaphase cells. Antibody production of each cell line was evaluated in batch cultures using Octet (ForteBio, Menlo Park, CA, USA).

## Results and discussion

### Determination of cell fusion efficiency

Cells stained with red and blue dyes were used to determine the efficiency of cell fusion. The blue- and red-stained cells were used as fluorescence minus one (FMO) controls for fluorochromes PE and BD Horizon V-450 in flow cytometry, respectively. Eliminating the background noise of possible unstained cells, the mean percentage of the fused cells emitting both red and blue fluorescence simultaneously was 18.5% 4.60% (n = 3). Considering that self-fusions among dye-stained cells are also likely to occur and could not be detected using a flow cytometer, the suggested fusion efficiency was at least 13.9%.

### Determination of ploidy

In a separate experiment, five single clones obtained from the PEG-treated cell population by ClonePix2, were randomly selected to determine the DNA ploidy using flow cytometry. From these data, it is evident that clone #1 was haploid compared to the diploid control (untreated) cells. While clones #2~#5 showed no significant difference in fluorescence intensity compared to the control group, the G2/M peaks of clones #2 and #3 appeared to be higher than that of control group. Given the fact that the DNA content of diploid G2/M is indistinguishable from that of tetraploid G0/G1 (DNA tetraploid), we proceeded to analyze metaphase cells of each clone to gain further insight into the chromosomes of the PEG-treated clones.

### Analysis of chromosome number

**Figure 1:**
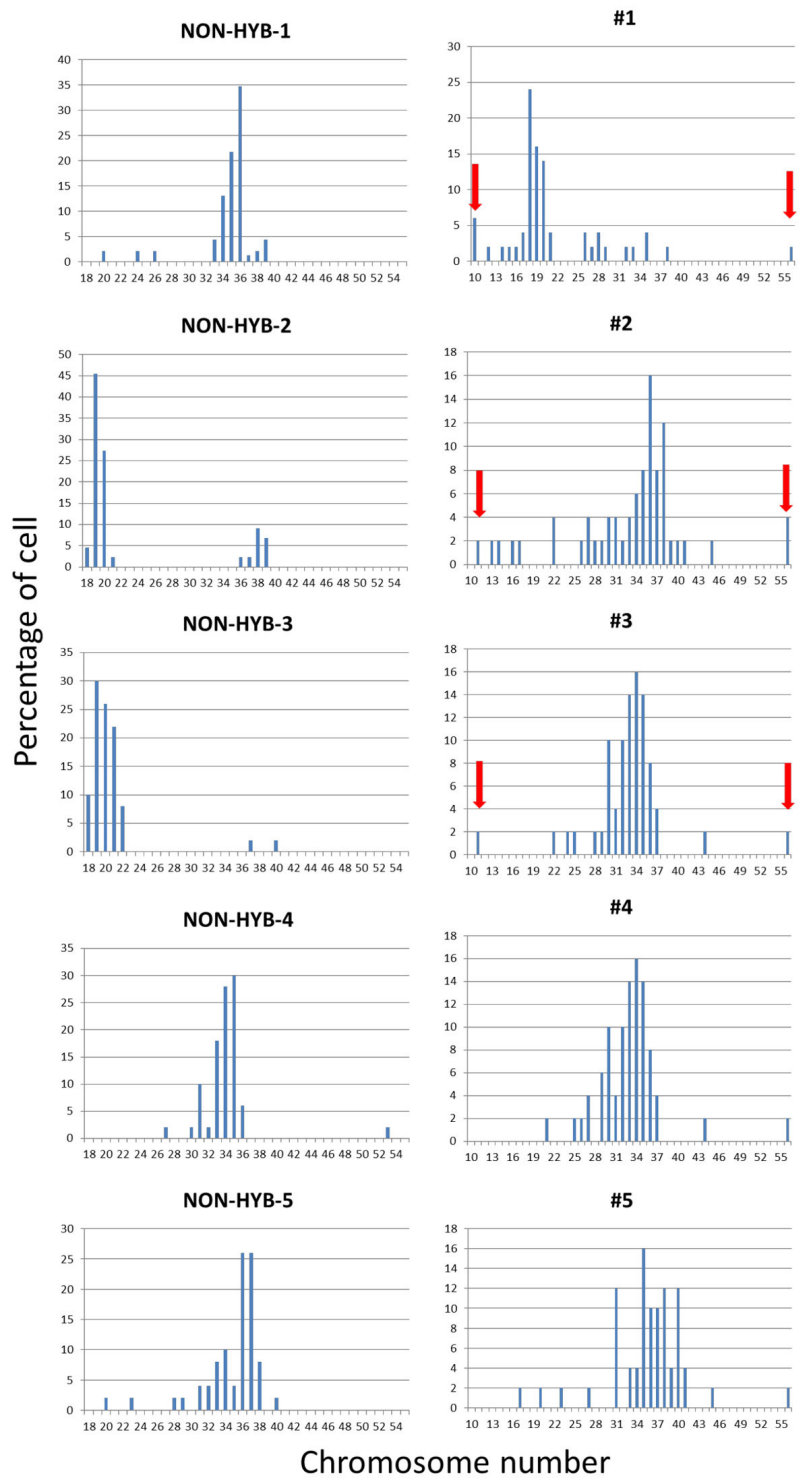
**Chromosome distribution of PEG-treated clones #1-#5 compared to control cell clones (NON-HYB-1- NON-HYB-5)**. Red arrows represent ambivalent chromosome numbers observed within the same cell line.

In the control group, approximately 60% possessed 26-30 chromosomes, two out of five clones contained 19-20 chromosomes, whereas the rest contained 35-36 chromosomes (n = 50). This shows that while the control group displayed aneuploidy to a certain extent, each clone maintained a certain number of chromosomes regardless of the number of passages and culture time. On the other hand, the PEG-treated clones did not exhibit any consistency in chromosomal number and a wide range of aneuploidy was observed (Figure 1). Notably, three treated clones (#1, #2 and #3), showed ambivalent chromosome numbers, i.e. cells with less chromosomes (<16) coexisted with cells having higher number of chromosomes (>41) (red arrows in Figure 1). These characteristics were not observed in the control group. Hence, we deduced that PEG-mediated cell fusion induced chromosomal instability that is different from the one occurring naturally.

### Evaluation of antibody concentration

The five cell lines obtained from PEG-treated group displayed distinct antibody-producing ability. Particularly, clones #1, #2 and #3, which exhibited ambivalent chromosome numbers, presented 1.82, 1.42 and 1.36 times higher final antibody concentration than the control cell pool, respectively. Specific antibody production rates of all these three clones were at least 1.57 times higher than that of control cell pool. Based on the fact that clones which exhibit ambivalent chromosome numbers showed higher final antibody concentration and production rate than the controls and PEG-treated clones #4 and #5, we suggest that diversity in chromosome number, a type of chromosomal instability induced by cell fusion is an essential factor in constructing high antibody-producing cell lines.

## Conclusions

The natural instability of chromosomes in CHO cells has often been an issue in establishing high-yielding cell lines for industrial use. This study has shown that high-producing cells developed by PEG-mediated cell fusion exhibit diversity in chromosomal number, a kind of chromosomal instability that does not occur naturally. This reveals a novel association between chromosomal instability and antibody production, and is expected to significantly contribute to the development of cell culture as well as construction of cell lines for industrial purpose in the future.

## Acknowledgements

This work was partly funded by a grant for the Project focused on developing key technology of discovering and manufacturing drugs for next-generation treatment and diagnosis from the Ministry of Economy, Trade and Industry of Japan and partly by a Grant-in-Aid for Scientific Research from the Japan Society for the Promotion of Science (JSPS) (No.26630433, 26249125).

